# Accuracy and Reliability of Computerized Surgical Planning Software in Anatomic Total Shoulder Arthroplasty

**DOI:** 10.7759/cureus.37400

**Published:** 2023-04-10

**Authors:** Yehia H Bedeir, Eloy Tabeayo, Te-feng A Chou, Konrad I Gruson

**Affiliations:** 1 Orthopedic Surgery, Montefiore Medical Center, Albert Einstein College of Medicine, Bronx, USA; 2 Orthopedic Sports Medicine and Shoulder Surgery, University of Alexandria, Alexandria, EGY

**Keywords:** 3d-planning, shoulder replacement, planning software, total shoulder arthroplasty, preoperative planning

## Abstract

Purpose: The primary purpose of this study was to assess the concordance between preoperatively determined implant parameters using CT-based planning software and surgically implanted prostheses. Secondarily, we sought to evaluate the agreement between preoperative plans performed by surgeons at different levels of training.

Methods: Patients with primary glenohumeral osteoarthritis who underwent anatomic total shoulder arthroplasty (aTSA) and had a preoperative CT scan according to Blueprint (Stryker, Mahwah, NJ) protocol to be used for preoperative planning were included. A cohort of short-stemmed (SS) and stemless cases performed between October 2017 and December 2018 was randomly selected from an institutional database for the study. Planning was performed separately by four observers at different levels of orthopedic training at a minimum of six months following the actual surgery. Concordance between the surgical decisions during planning and the actually utilized implants was calculated. Additionally, inter-rater agreement was analyzed using the intra-class correlation coefficient (ICC). Implant parameters assessed were glenoid size, backside radius of curvature, and the need for posterior augment, in addition to humeral stem/nucleus size, head size, head height, and head eccentricity.

Results: Twenty-one patients were included (10 stemmed and 11 stemless) with a cohort comprising 12 (57%) females with a median age of 62 years (IQR 59.5,67). There was a total of 544 decision possibilities based on the above parameters. The total number of decisions that matched surgical data was 333 (61.2%). Prediction of glenoid component augmentation need and size was the variable that matched most with surgical data (83.3%), whereas nucleus/stem size was the worst (42.9%). Interobserver agreement was excellent in one variable, good in three variables, moderate in one, and poor in two. The best interobserver agreement was with regard to head height.

Conclusion: Preoperative planning using CT-based software may be more accurate for the glenoid component when compared to humeral-sided parameters. Specifically, planning may be most helpful in determining the need and the size of glenoid component augmentation. Utilizing computerized software demonstrates high reliability, even among surgeons early in their orthopedic training.

## Introduction

Anatomic total shoulder arthroplasty (aTSA) is a well-accepted treatment option for advanced arthritis of the glenohumeral joint with an intact rotator cuff following failure of non-surgical management [1]. The implementation of preoperative templating and patient-specific instruments have grown in popularity with the goal of improving implant positioning and, thereby, patient-reported outcomes [2]. Traditionally, plain radiographs and 2D CT scans were the main imaging modalities used for preoperative assessment of glenoid wear, version, size, and inclination, in addition to humeral head size and bone quality [1]. Computerized planning programs using computerized tomography (CT) with 3D modeling have been recently developed for a more accurate assessment of these parameters and to provide automated values for glenoid measurement indices [3]. This can assist the surgeon in the selection of implants by trialing them virtually preoperatively and optimizing size and fit. CT scans have been shown to result in a significant change in the preoperative plan for addressing glenoid wear in advanced glenoid wear patterns when compared to evaluation using axillary radiographs alone [4,5]. Glenoid loosening persistently remains the most common reason for the failure of aTSA [6,7]. Several factors, such as glenoid component malposition, incomplete correction of the bone pathological conditions, and persistent humeral head subluxation, have been associated with glenoid loosening [2]. The glenoid version can be restored by eccentric reaming, use of bone graft, or placement of augmented glenoid components [8]. Choosing the appropriate sizes of different components of TSA can help minimize the risk of intraoperative fractures, postoperative instability, overstuffing of the joint, and impingement on the rotator cuff [1]. Moreover, recent studies have shown a significant reduction in the number and cost of sterilized trays when preoperative planning was applied [9].

Despite the improved accuracy of preoperative planning using CT, surgeons still often modify the predetermined implant sizes and placement according to intraoperative findings. The concordance between the implant selected using the preoperative planning software and the implant placed during surgery has not been well-defined in the existing literature [10-14]. Reschenbacher et al. retrospectively reviewed 50 cases of stemless aTSA performed by two surgeons and found that there was perfect concordance between the preoperative plan and the actual implants in only 11 cases (22%). They found that the glenoid component demonstrated the highest concordance when compared to the humeral head and nucleus [12]. Similarly, Raiss et al. reported an 85% complete concordance between the size, backside radius, and need for augments in the surgically implanted glenoid compared with the preoperatively planned implant [10]. The authors reported that 58/60 cases of planned posterior augments were implanted as such and that there was a 98% concordance between planned and implanted augment size. Finally, Parsons et al., in a study assessing inter- and intra-surgeon variability in glenoid component planning in aTSA, found significant variability with regard to the need for posterior augments and the ideal correction of glenoid retroversion [14]. With regard to planning for humeral stem size, Wittmann et al. found that the concordance between the size implanted, and the preoperatively planned size was 44.2% for short uncemented stems, though the planned size was within one size from the actual stem in nearly 88% of cases [11]. The reason for the reported differences between planned component sizes and the actual implanted components may have to do with the lack of universally accepted ideal component positioning, in addition to the tendency of surgeons to follow the surgical plans that were performed just prior to surgery.

The purpose of the current study was to 1) assess the concordance between ideal implant size and positioning using planning software and actual intraoperatively implanted prostheses for both SS and stemless aTSA and 2) evaluate the correlation between preoperative plans performed by surgeons at different levels of training. Our hypothesis was that there would be an inverse correlation between preoperative plans by trainees as compared to experienced shoulder surgeons and that there would be high concordance between implants selected using commercially available preoperative planning software and implants utilized during surgery.

## Materials and methods

This was a retrospective study performed on a random selection of patients who underwent aTSA at a single institution by a single fellowship-trained shoulder surgeon from October 2017 to December 2018. The study was approved by the institutional review board at the Albert Einstein College of Medicine (IRB #2021-13367). The inclusion criteria were adult patients: 1) with non-inflammatory glenohumeral arthritis; 2) who underwent stemmed or stemless aTSA; 3) whose glenoid was classified as a Walch A- or B-type; and 4) who had a preoperative CT scan formatted according to the Blueprint (Stryker, Mahwah, NJ) protocol for planning purposes. The glenoid deformity was determined by consensus between the two senior authors. Patients with incomplete records regarding implant sizes utilized during surgery and those with inadequate preoperative imaging were excluded. Furthermore, any patients who required humeral component cementing, those with C- or D-type glenoid morphology, and those with the prior surgical intervention were excluded from the study.

Preoperative planning and surgical data gathering

Preoperative CT scans were retrieved, and guidelines for implant selection and positioning were distributed among four investigators at different levels of training: a second-year resident, a shoulder fellow, and two fellowship-trained shoulder surgeons. At the time of the study, one of the fellowship-trained surgeons was in practice for <5 years and the other for >10 years. The Blueprint (Stryker, Mahwah, NJ) commercially available software was used to plan these surgeries. Blueprint (Stryker, Mahwah, NJ) allows the surgeon to predict sizes of humeral stem/nucleus, humeral head and glenoid size based on preoperative CT scan. Additionally, head height, head eccentricity, glenoid backside radius for non-augmented glenoid, and the need for and size of augmented glenoid components were determined. Participants each had experience using the planning software at least five times previously and sought to achieve general sizing and implant orientation parameters determined at the start of the study: for the glenoid, maximum seating with minimal bone resection to achieve placement in native inclination, or within 0-10 degrees superior if deformity presents, in addition to version within 0-10 degrees retroverted; for the humeral head, the superior aspect of the articular margin within 6 mm of the top of the greater tuberosity with minimal to no calcar underhang. With regards to the humeral stem component, we sought to achieve a distal fill ratio (DFR) of approximately 70% for the short stem [15] and a stable nucleus without lateral perforation for the stemless implants, after placing the component in the anatomic humeral retroversion. Of note, only centered (non-eccentric) humeral head options are available for stemless cases. Each of the investigators recorded their selected glenoid component size, polyethylene backside radius of curvature and augment, if applicable, as well as their preferred head size and height, and stem or nucleus size for stemmed or stemless implants, respectively.

Options that were available for surgeons to select for each variable are shown in Table 1. All four observers predicted each of the variables on the included patients, with the exception of head eccentricity which was an option only with SS implants. The observers made a total of 544 decisions (136 for each observer) during the planning. In an effort to minimize the bias of strictly adhering to the computerized surgical plan during intraoperative implant selection, we elected to re-plan the cases at a minimum of six months following the surgery using the above ideal positions. The results of the re-planning were then compared with the actual implanted components to determine concordance. Each observer was told whether a SS or stemless humeral component was utilized for the case so appropriate planning for the humeral stem or nucleus, respectively, could be carried out.

**Table 1:** Implant parameters available for surgeons during planning

Variable	Available size options
Nucleus (stemless)	1, 2, 3
Stem (SS)	1, 2, 3, 4, 5, 6, 7, 8
Head size	37 to 56
Head height	Stemless 16, 18, 21; Stemmed 17, 20, 23
Head eccentricity	Low (1.5); High (4)
Glenoid size	Small, Medium, Large, Extra-large
Glenoid backside radius for non-augmented glenoids	Small: 30, 35, 40; Medium: 30, 35, 40; Large: 40, 50, 60; Extra-large: 40, 50, 60
Glenoid augment wedge size	0, 15, 25, 35 half wedges

Surgical technique

The surgical technique was performed in a standard fashion in each case with a free-hand anatomic neck osteotomy for the stemless implants and use of an intramedullary cutting block for the SS humeral implants. A standard deltopectoral incision with a subscapularis peel was used in every case with repair through bone tunnels in the lesser tuberosity using #2 non-absorbable sutures. The surgery was performed under either general anesthesia alone or general anesthesia combined with regional anesthesia. None of the humeral components were cemented. Among the included cases, there were no changes of surgical plan from a stemless component to a SS component based on metaphyseal bone quality. Our standard practice has been to use fluoroscopy during the cases to ensure appropriate sizing and positioning of the implants, particularly on the humeral side.

The Simpliciti stemless implant was utilized for all of the stemless cases (Wright Medical, Memphis, TN, USA). The SS component was the Ascend Flex with proximal porous coating in all cases (Wright Medical, Memphis, TN, USA). Prior to May 2018, only uncemented SS humeral components were utilized and after May 2018, only the stemless implant was utilized. The glenoid components were hybrid with a press-fit polyethylene central peg with three peripheral cemented pegs, with and without posterior augmentation (Aequalis Perform Cortiloc or Perform +, Wright Medical, Memphis, TN, USA). Charts of the included patients were then retrieved to extract the types and sizes of implants that were actually inserted during surgery, in addition to demographic data.

Statistical analysis

Given the non-parametric nature of the data, continuous variables were presented as a median and associated inter-quartile range (IQR). The standard chi-square test was utilized to evaluate categorical variables. Fisher’s exact test was utilized to compare the association between glenoid morphology and the need for augmentation given the lack of augments used in cases of A1, A2 and B1 glenoids. Agreement between each observer and surgical data was described using number and percentage. Intraclass correlation coefficient (ICC) was used to assess interobserver agreement between the four observers. The ICC classification suggested by Koo and Li was utilized: a value below 0.50 described poor strength of agreement; between 0.50 and <0.75, moderate agreement; between 0.75 and <0.90, good agreement; and >0.90, excellent agreement [16]. A p-value <0.05 was considered significant. Statistical analysis was performed using IBM SPSS software package version 20.0.

## Results

A total of 21 patients were included, of which 12 (57%) were females with a median age of 62 years (IQR 59.5,67). The median body mass index (BMI) for the cohort was 30.2 kg/m^2^ (IQR 26.9,34.9) and surgery involved the right shoulder in 14 cases (67%). Of the total cases, 10 were SS aTSA and 11 were stemless aTSA. Preoperative glenoid morphology was A1 in six (28.6%), A2 in one (4.8%), B1 in three (14.3%), B2 in six (28.6%), and B3 in five (23.8%). There were six cases that utilized a posteriorly augmented glenoid. Glenoids with either B2 or B3 morphology were significantly more likely to have an augmented glenoid placed (p=0.01). The overall concordance between ideal planning by each observer for each parameter and surgical data ranged from 58.8% to 64% (Table 2).

**Table 2:** Agreement between observers’ predictions and surgical data Number (percent) of observers’ predictions that matched surgical data

	Observer 1, n (%)	Observer 2, n (%)	Observer 3, n (%)	Observer 4, n (%)	Overall concordance, (%)
Nucleus / Stem	9 (42.9)	12 (57.1)	10 (47.6)	5 (23.8)	42.9
Head size	7 (33.3)	13 (61.9)	14 (66.7)	15 (71.4)	58.3
Head height	11 (52.4)	12 (57.1)	11 (52.4)	11 (52.4)	53.6
Head eccentricity (10)	10 (100)	7 (70)	2 (20)	6 (60)	62.5
Glenoid size	12 (57.1)	13 (61.9)	12 (57.1)	14 (66.7)	60.7
Non-augmented glenoid backside radius	14 (66.7)	11 (52.4)	16 (76.2)	16 (76.2)	67.9
Augmented glenoid size	17 (81.0)	17 (81.0)	16 (76.2)	20 (95.2)	83.3
Total decisions (136)	80 (58.8)	85 (62.5)	81 (59.6)	87 (64.0)	333 (61.2)

The total number of decisions that matched surgical data was 333 (61.2%). Concordance between surgical data and the overall mean of all observers for each variable ranged from 42.9% to 83.4%. Prediction of glenoid component augmentation need, and size was the variable that matched most with surgical data (83.3%), whereas nucleus/stem size was the worst (42.9%). Prediction of only a single variable, humeral head eccentricity, by one observer matched surgical data perfectly (100%). With regards to the humeral stem size, 37.5% demonstrated perfect concordance with the actual implant, 32.5% differed by one size, 27.5% differed by two sizes and 2.5% differed by three sizes. For nucleus planning, 47.7% demonstrated perfect concordance and 100% of decisions were within one size of the actual implant. The difference between the nucleus and humeral stem for perfect concordance was not significant (p=0.47). The decisions for humeral head size were concordant in 58.3%, were within one size in 33.3%, within two sizes in 7.1% and within three sizes in 1.1%. Concordance was significantly higher among the humeral diameter decisions in stemless implants versus SS implants (72.7% vs. 42.5%, p=0.005). The decisions for the humeral height were concordant in 58.3% of cases, were within one size in 33.3% of cases, and were within two sizes in 8.3% of cases. Of the discordant decisions, 80% (28/35) were planned larger than the actual implants. The concordance with regards to the glenoid component is given in Table 3. Concordance of decisions regarding the humeral head were significantly lower than those for the glenoid (57.2% vs. 73.3%, p=0.002). The difference in concordance between humeral and glenoid components increased when also considering the sizing of the nucleus/stem (54.8% vs. 73.3%, p=0.00002).

**Table 3:** Concordance of glenoid component between planned and actual implant

Implant Concordance	Glenoid Size (%)	Non-augmented Need/ Backside Radius (%)	Glenoid Augmentation Need/Size (%)
Total Concordant	60.7%	67.9%	83.3%
Total Discordant	39.3%	32.1%	16.7%
Larger than planned			
Within 1 size	10.7%	16.6%	2.4%
Within 2 sizes	0.0%	6.0%	0.0%
Within 3 sizes	0.0%	0.0%	0.0%
Smaller than planned			
Within 1 size	28.6%	8.3%	13.1%
Within 2 sizes	0.0%	0.0%	1.2%
Within 3 sizes	0.0%	1.2%	0.0%

When interobserver agreement was analyzed, agreement was excellent in one variable, good in three variables, moderate in one and poor in two. The best interobserver agreement was in head height. Agreement between observers is shown in order of strength in Table 4.

**Table 4:** Interobserver agreement for measured variables ICC: Intraclass Correlation Coefficient; CI: Confidence interval

	ICC coefficient	95% CI	Level of agreement
Head height	0.989	0.979 – 0.995	Excellent
Head size	0.849	0.735 – 0.928	Good
Glenoid size	0.819	0.688 – 0.912	Good
Glenoid augmentation	0.782	0.633 – 0.892	Good
Nucleus / Stem	0.713	0.536 – 0.854	Moderate
Glenoid backside radius	0.028	-0.119 – 0.270	Poor
Head eccentricity (10)	0.188	-0.072 – 0.596	Poor

## Discussion

The major findings of this study were that the use of preoperative planning software for aTSA demonstrated good concordance with actually implanted components with regard to glenoid-related parameters, particularly for the need and size of augmentation. The concordance was lower with humeral-sided parameters. Furthermore, there is a high degree of reliability with regard to the use of preoperative planning software, even among orthopedic trainees.

Computerized planning software that utilizes a preoperative CT scan has recently become popularized for both aTSA and rTSA [9-12,17]. Posteriorly augmented glenoid components are used to correct glenoid retroversion or erosion while preserving the native bone without the need for extensive eccentric reaming. Favorable clinical and radiographic outcomes have been reported with posteriorly augmented glenoids in patients with Walch B2 or B3 glenoid deformity [18-20]. We found that only 61.2% of surgeons’ predictions matched with surgical data, though decision concordance for glenoid-related parameters was significantly higher than for the humeral side. Our findings were considerably lower than previously reported for aTSA [10-12,14]. Raiss et al., in a study examining concordance between preoperative planning and implanted glenoid components, reported 85% complete concordance between planned and implanted glenoids [10]. In 96% of cases, the same glenoid size was utilized, and in 91%, the planned backside radius was. There was a 98% concordance between the planned size of the posterior augmented to that implanted. We also found a high concordance between the need for and size of the posterior glenoid augments with a mean of 83.3% between observers. In our study, an additional 15.5% of the cases were within one augment size. Regarding glenoid size, 61% were concordant with a plan in our study with all discordant cases within one size. The range of deviation of the discordant glenoid sizes in our study was similar to that of Rechenmacher et al. [12]. Our study differed from that of Raiss et al. in that we re-planned our cases at least six months following the actual procedure to minimize the bias inherent in performing the surgery based on adhering to the surgical plan. Furthermore, we included the observations from trainees and provided each observer with some guidelines with regard to implant size and positioning. Parsons et al. reported significant inter- and intrasurgeon variability with regard to glenoid augment use and size, though the degree of inclination and version achieved was low [14]. The authors concluded that orthopedic surgeons likely have different thoughts regarding the optimal reconstruction of the glenoid, and that different implant designs and augment use could be used to achieve a desired implant position and a successful clinical outcome. 

We found that concordance between the planned humeral stem component and actual implants was low, with an average of 42.9% among the observers. Additionally, we found that the discordance in the humeral components was significantly higher than for the glenoid components. The latter finding was consistent with that reported by others [12]. Rechenmacher et al. studied a cohort of patients undergoing only stemless aTSA and found that nucleus and humeral head concordance were 60% and 38%, respectively. They found that 100% of the nucleus sizes were within one size of the implanted one. We found the concordance of the nucleus size at 53% and 100% were within one size of the actual implant. With regards to the short stems, we found a concordance of 38% within 70% falling within one size of the actual. Our results are similar to those of Wittmann who found concordance of the short stem at 44% with 88% falling within one size of the actual [11]. With regard to the specific implant used in our study, the metaphyseal bone is impacted as opposed to removed, potentially leading to the size discrepancy. Furthermore, it is difficult to assess bone quality from preoperative imaging. Of the deviations in the current study, for instance, 72% were oversized compared with the implanted stem. Certainly, in light of the potential for stress shielding when using larger stems [15], care should be taken to use the smallest stem necessary to achieve stable fixation. 

Despite the different levels of training among the participating surgeons in this study, intersurgeon agreement between the plans was good or excellent in more than half of the variables reviewed. The variability among observers was highest with regard to the glenoid backside radius and the amount of humeral head eccentricity for the SS implants. Parson et al., in a study assessing the reliability of glenoid implant type (augment versus no augment) and augment size among nine surgeons, found a high degree of intersurgeon variability with regard to those parameters [14]. In contrast, we found a good intersurgeon correlation between the observers with regard to the need and size for glenoid augmentation. The difference may lie in the number of surgeons used in their study, in addition to the position parameters defined prior to the planning in the current study. Furthermore, we did not evaluate the amount of correction achieved with regard to inclination and version, and a different implant system was utilized. 

This study certainly has several limitations that bear mention. This is a retrospective study that was performed on a small, randomly selected patient sample. Additionally, the outcomes of this study may not be generalizable to all commercially available shoulder arthroplasty implants, as only the Blueprint planning software and one implant system were used for all cases. Waltz et al. recently demonstrated that the region of the glenoid from which version and inclination were measured differed among two widely used preoperative planning software systems [21]. Moreover, one of the participating observers was the main surgeon who actually operated on these patients. This might have introduced bias as the operating surgeon may tend to adhere to their preoperative plan despite intraoperative findings. In order to minimize the potential for bias, all of the selective cases were re-planned at least six months following the actual procedure and compared to the surgical plan.

## Conclusions

Preoperative planning may be important in assessing the general size and positioning of components needed during aTSA, though intraoperative decision-making still plays a major role in ultimate implant selection. In particular, planning software seems to be most helpful regarding the need for and size of posteriorly augmented glenoid components. Even when the actual implants deviated from the preoperative plan, most were within one size of the implanted component. Planning software for aTSA can reliably be used among surgeons, even those early in their training.
